# Research Participant Selection Bias in the Workshop Using Socially Assistive Robots for Older Adults and Its Effect on Population Representativeness

**DOI:** 10.3390/ijerph20105915

**Published:** 2023-05-22

**Authors:** Toshiharu Igarashi, Ikuko Sugawara, Takenobu Inoue, Misato Nihei

**Affiliations:** 1 Department of Human and Engineered Environmental Studies, The University of Tokyo, Kashiwanoha 5-1-5, Chiba 277-8563, Japan; 2 Faculty of Service Management, Bunri University of Hospitality, 311-1, Kashiwabara-shinden, Sainatama 350-1336, Japan; 3 Research Institute of National Rehabilitation, Center for the Persons with Disabilities, 1, Namiki 4-chome, Saitama 359-8555, Japan; 4 Institute of Gerontology, The University of Tokyo, 3-1, Hongo 7-chome, Bunkyo-ku, Tokyo 113-8654, Japan

**Keywords:** socially assistive robots, selection bias, population representativeness, human-robot interaction, LSNS-6

## Abstract

Every research participant has their own personality characteristics. For example, older adults assisted by socially assistive robots (SAR) may have their own unique characteristics and may not be representative of the general population of older adults. In this research, we compared the average personality characteristics of participants in a workshop on robotics recruited directly through posting with those of older Japanese adults to examine participant selection bias and group representativeness for future study of SARs. After a one-week recruitment period, the workshop was attended by 20 older participants (nine males and 11 females) aged between 62 and 86 years. Extroversion among workshop participants was 4.38, 0.40 higher than the average for older adults in Japan. The workshop participants’ openness was 4.55, 1.09 higher than the average for the Japanese elderly. Thus, the results indicate a slight selection bias in the personal characteristics of the participants depending on the recruitment method when compared to the Japan national average for older adults. In addition, only one of 20 participants was below the cutoff on the LSNS-6 score and considered to have a tendency toward social isolation. The development and introduction of socially assistive robots is often being considered to support people in social isolation in their daily lives; however, the results of this study showed that it is difficult to recruit people who tend to be socially isolated when gathering research participants by methods such as posting. Therefore, the effectiveness of the method of recruiting participants should be carefully verified in research regarding socially assistive robots.

## 1. Introduction

The lifestyles of Japanese older adults are changing. For instance, two or three families living together was commonplace in the past. However, in recent years, an increasing number of Japanese households comprise single adults and married older adults [1]. Therefore, care for older persons has become a significant issue in Japan. To meet the demand for 2.45 million care workers by the fiscal year 2025, 60,000 additional care workers are needed [2]. The shortage of care workers in Japan has been a serious and prolonged problem. To address this problem, some have suggested interventions via socially assistive robots (SARs) specially designed for older adults [3]. Research shows that appropriate interventions using SARs can help prevent the progression of cognitive decline and decrease depression among older adults [4,5]. However, there are large individual differences in usage and retention rates; furthermore, despite proper introduction, inappropriate use has been reported [6].

Importantly, based on the opinions of older adults, four issues related to the use of SARs have emerged: (1) the SAR’s roles; (2) its appearance; (3) the normative/ethical issues in its use in care for older persons; and (4) the interaction between an older adult and a SAR. The last issue can be further subdivided into the technical and human aspects of this interaction [7]. 

While most of these issues have been investigated intensively, few have examined the human aspect of the interaction between SARs and older adults. SARs are defined as “the intersection of AR and SIR” [8]. While SARs are similar to assistive robotics in that both assist human users, the former differs from the latter in the sense that this assistance occurs through social interaction. However, as important as interpersonal relationships are, understanding the characteristics of older adults using SARs is also a very important research subject. Up to this point, research has typically focused on gender as the main personality attribute of research participants that affects the outcome of SAR intervention experiments [9]. To address this research gap, this study examines participant selection bias and population representativeness by comparing the personality characteristics of participants recruited directly by posting and indirectly by recommendations of nursing home staff for intervention experiments with SARs in Japan. 

## 2. Literature Review

### 2.1. Study Design and Bias

Intervention studies using SARs fall into three categories: randomized controlled trials (RCTs), crossover studies, and uncontrolled studies. Notably, most studies on SARs are uncontrolled, with only a few RCTs and crossover studies.

Biases affecting these intervention studies include selection, information, and confounding. To address selection bias, researchers need to control for the attributes of study participants according to the study design [10]. Martinson reported that research programs targeting older adults have limited reach and may have substantial volunteer bias [11]. As conducting an exhaustive survey is not always practical, researchers should carefully consider whether the sampled participants are representative of the population.

### 2.2. Relationship between Personality and Participant Seletion Bias

Personality refers to “the characteristics of a person that describe a consistent pattern of feelings, thoughts, and behaviors” [12]. Among recent theories of personality traits, the Big-Five Inventory [13,14] and the Five-Factor Model of Personality [15] are prominent theories with extensive evidence. One commonality is that both models view personality within five broad frameworks or attributes: extroversion, agreeableness, conscientiousness, neuroticism, and openness. For instance, on the relationship between personality and bias, Dollinger noted that an individual’s agreeableness and openness characteristics influence volunteer bias [16].

Moreover, one widely used scale for measuring personality, the Ten Item Personality Inventory (TIPI) created by Gosling, Rentfrow, and Swann, is based on the Big Five Inventory. Specifically, the TIPI measures the Big Five personality attributes [17]. It has been widely used in fields such as social psychology, political psychology, and behavioral economics [18,19]. Here, we used the Japanese version of the TIPI, created by Koshio et al. [20], to survey the personality of the study participants. The validity and reliability of this version of the scale have been demonstrated by Iwasa et al. [21].

Note that, depending on an older adult’s personality, their relationship and involvement with the care facility staff may differ substantially. This can influence their tendency to participate in intervention studies. Thus, both their personality traits and relationships may differentiate them from the representative population. However, to the best of our knowledge, few intervention studies on SARs conduct experiments while accounting for the effects of bias caused by such individual personality traits.

### 2.3. Study Motivation

Two main methods are used for recruiting participants for HRI studies of older adults: (1) direct recruitment by the research team, such as posting flyers and soliciting participants via e-mail; and (2) the research team requests the senior living/care facility and staff to solicit participants, and they select participants indirectly. As noted above, participants’ relationships with staff can bias their tendency to participate in experiments.

We have developed a robot study for nursing homes and serviced apartments. However, the “staff” factor may have introduced a bias in participant selection in the cases where participants were indirectly recruited by staff.

**Figure 1:**
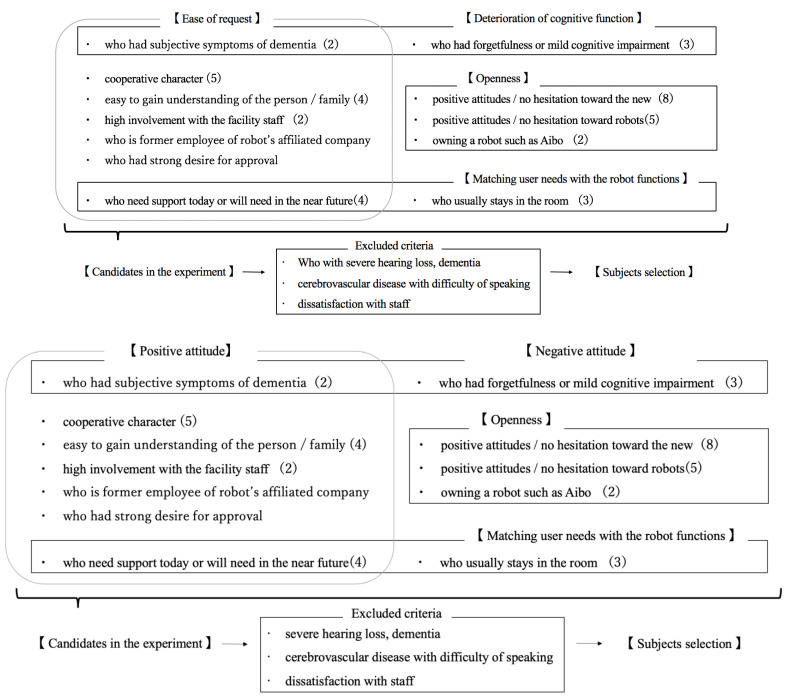
Care staff selection criteria and their inclusive concepts from 13 people interviewed. Care staff selected candidates based on “ease of request”, “openness”, “deterioration of cognitive function”, and “matching user needs with robot functions”. However, if any of the “exclusion criteria” were met, the person was removed from the experimental participants [22]. (© Igarashi 2019).

In fact, Igarashi et al. [22] interviewed 13 staff members (five men and eight women) involved in the selection of study participants for SAR-based intervention experiments over the past 10 years at six elderly care facilities (Figure 1). The interview guide included five interview items: (1) basic information about the interviewees, (2) basic information about the selected subjects, (3) subject selection criteria, (4) relationship with the selected subjects, and (5) work-related psychological burden scale. The results showed that the subjects were proactively selected from the elderly who were easy to request, such as those with “cooperative personalities” and “high involvement with facility staff”, as well as those with high openness tendencies, such as interest in new things and robots.

Furthermore, the personality characteristics of the study participants who were selected by the facility staff were examined and found to be 4.78 for extraversion, 5.78 for agreeableness, 4.72 for conscientiousness, 3.56 for neuroticism, and 4.33 for openness. In a previous study by Iwasa and colleagues, the national mean scores for Japanese women aged 80 to 84 (*n* = 49) were 3.95 for extroversion, 5.63 for agreeableness, 4.37 for conscientiousness, 4.13 for neuroticism, and 3.46 for openness. Therefore, it can be seen that the above follow-up subjects were selected for their high extroversion, openness, and low neuroticism tendencies.

Although experiments have been conducted to introduce SARs to users of such facilities, there has been no in-depth discussion of the criteria for selecting study participants. Therefore, in this study, we will investigate the personality characteristics of HRI research participants in the cases of (1) direct recruitment and (2) selection of research participants by facility staff, based on the Big Five characteristics of the research participants. We will also clarify the relationship between the personality of the research participants recruited in the case of direct recruitment and the representativeness of the elderly population in Japan.

## 3. Method

First, the researchers recruited participants by mail in May 2019 to conduct a workshop on SARs. Participants were recruited by posting to senior citizens living in the area through a town-supported non-profit organization. One week before the workshop was to be held, we mailed a notice to 100 households where senior citizens lived in the town.

The target audience was men and women aged 60 or older living in I town who were not residing in a senior care facility. he workshop was held in the conference room on the first floor of the community center for the recruited participants.

**Figure 2:**
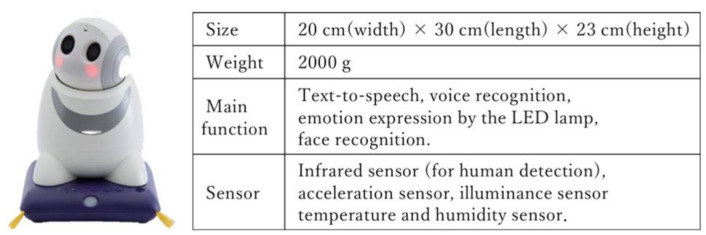
Size, weight, main functions, and mounted sensors of “PaPeRo” [27] (© Copyright © NEC Platforms, Ltd. 2014–2023).

In the workshop, we explained current problems in the daily lives of older adults, gave an overview of lifestyle support robots, demonstrated their current functions, collected opinions on desired functions for lifestyle support robots in future development, and held discussions among participants. At the end of the workshop, a robot was introduced called PaPeRo (Partner-type-Personal-Robot) as a concrete example. This robot is equipped with speech recognition, speech synthesis, facial image recognition, autonomous movement, head movement, light indication function, and tactile sensors (see Figure 2 for a brief overview of dimensions and features).

After the workshop, a survey was conducted using a self-administered questionnaire. The questionnaire included items related to five categories: (1) participants’ personality characteristics (7-point Likert scale, 10 questions); (2) their impressions of the life support robots introduced at the workshop (7-point Likert scale, eight questions); (3) their willingness to participate in a study to introduce a life support robot in their homes (5-point Likert scale, one question); (4) LSNS-6 (six questions); and (5) problems in daily life.

The LSNS-6 is a shortened version of the Lubben Social Network Scale, an international scale widely used for screening social isolation in the elderly [23]. The cutoff value is considered less than 12 points, and the percentage of social isolation among the elderly in Japan is reported to be 38.0% [24,25].

As for “(2) the impressions of the life support robots”, eight items were set, referring to the study by Kamide et al. [26] (Table 1).

## 4. Results

After a weeklong recruitment period, the workshop was attended by 20 older participants (nine males and 11 females) aged between 62 and 86 years. The mean age of the participants was 74.55 years with a standard deviation of 5.54.

Regarding the number of people living together, two respondents lived alone (no cohabitation), eight had one person living with them, six had two people living with them, two had three people living with them, one had four people living with them, and one had six people living with them

As for difficulties in daily life to which multiple answers were given, four respondents answered “None”, nine respondents answered “Health”, six respondents answered “Transportation (including outdoor shopping)”, and two respondents answered “Household chores and daily life support (not involving outdoor transportation)”.

The Big-Five personality traits of the participants were assessed on a 7-point scale. The average values of extroversion, agreeableness, conscientiousness, neuroticism, and openness were 4.38, 5.48, 4.43, 3.13, and 4.55, respectively. The average impression value of the communication robot was 5.14 on a 7-point scale.

Regarding interest in the experiment of introducing a robot into the home, the average score was 3.30 on a 5-point scale, with a standard deviation of 0.92. As for the impression of the value of the communication robot, the mean was 5.14 on a 7-point scale, with a standard deviation of 0.38. The LSNS registered a mean of 17.65 with a standard deviation of 3.77.

## 5. Analysis

### 5.1. Comparison between Workshop Participants and Japanese Population Averages

First, we examined the relationship between personality, selection criteria, and frequency of involvement. Iwasa et al. reported the following national average scores for Japanese women aged 80 to 84 (*n* = 49): 3.95 for extraversion, 5.63 for agreeableness, 4.37 for conscientiousness, 4.13 for neuroticism, and 3.46 for openness. By comparison, the workshop participants reported higher values for extroversion (higher by 0.43), diligence (0.06), and openness (1.09). Conversely, they reported lower values for cooperativeness (lower by −0.15) and neuroticism (−1.00).

### 5.2. Comparison of Direct and Indirect Sampling Techniques

Comparing workshop posting recruitment with nursing home staff recruitment, staff recruited more extroverted participants (difference = 0.40). This result is also consistent with the GTA analysis (“more conversations”). Conversely, more open (difference = 0.22) and less neurotic (difference = 0.43) candidates were recruited through workshop recruitment than by nursing home staff (difference = 0.22). Notably, both methods recruited more extroverted (higher by 0.43), more open (higher by 0.87), and less neurotic participants (lower by 0.57).

**Figure 3:**
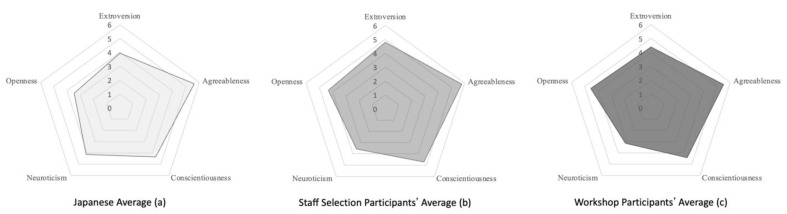
Visual score comparison among Workshop Participants, Staff Selection Participants, and National Average.

Regarding cooperativeness, both workshop and nursing home study participants fell within a range of ±0.15 compared to the Japanese average. However, this may be due to the high average cooperativeness among the Japanese population. Meanwhile, diligence was almost the same as the Japanese average in the case of workshop recruitment, but 0.35 higher for nursing home staff recruitment (Table 2 and Figure 3).

### 5.3. Correlation Coefficients between Personality Traits and Willingness to Participate in Experiments among Workshop Participants

Next, we examined the correlation between participants’ willingness to participate in the experiment with their five personality traits and their impression value of the robot (Table 3). The correlation coefficient with the impression value of the communication robot was 0.38, indicating that there may have been some correlation. Meanwhile, the correlation coefficients between the five personality traits and willingness to participate in the experiment were all less than 0.3, suggesting that there was a limited correlation. However, none of the workshop participants responded they were “1: not at all interested” in participating in the study; all responded “5: very interested” to “2: not very interested”. As mentioned above, it is possible that the population which participated in the workshops was originally a group of older adults who were highly extroverted and open-minded, making it difficult to correlate the results.

### 5.4. Number of People Living Together

Two respondents were living alone (no cohabitation), eight had one person living with them, five had two people living with them, two had three people living with them, one had four people living with them, and one had six people living them. 

Two respondents lived alone (no cohabitation), accounting for 10% of the workshop participants. There were eight people living alone, but excluding those living with siblings or friends, there were six married-couple-only households, which accounted for 33.3% of the workshop participants.

Compared to Japanese national statistics, which show that 28.8% of elderly households live alone and 32.3% live in couple-only households, we were able to gather the same number of couple-only households but were unable to gather participants who only live alone as close to the population.

### 5.5. Social Isolation and Population Representativeness

Although an LSNS score of 12 or below is considered socially isolated, only one of the 20 participants in the workshop fell below the cutoff. According to a previous study, 38% of elderly Japanese are socially isolated, and it is difficult to recruit people who are considered socially isolated to participate in a study by posting information about the study. In particular, socially assistive robots are often identified by the label, “for elderly people who live alone or are depressed”, but further outreach research is needed to verify whether they are truly effective for such target populations.

## 6. Discussion

### 6.1. Extroversion and Openness

Studies introducing socially assistive robots often examine changes in the quantity and quality of communication. However, it is known that human communication style is influenced by extroversion and interest in new things by openness.

Extroversion among workshop participants was 4.38, 0.40 higher than the average for older adults in Japan. The workshop participants’ openness was 4.55, 1.09 higher than the average for the Japanese elderly. The same trend was observed in the case of the facility staff selection, where the extroversion of the staff-selected participants was 0.83 higher than the average, and their openness was 0.87 higher than the average.

Thus, the results indicate a slight selection bias in the personal characteristics of the participants depending on the recruitment method when compared to the national average. Therefore, even if good results are obtained in a study using similar methods for recruiting research participants, it is necessary to carefully discuss whether the same results can be obtained for the population (users across countries and cultures). The best way to deal with the sampling problem is to conduct large-scale random sampling (RCT). However, RCT is often difficult due to time and financial constraints, therefore stratified analysis by personality traits can instead be conducted with a sample size as large as possible.

### 6.2. Outreach to Social Isolation

Only one of 20 participants was below the cutoff on the LSNS-6 score and was considered to have a tendency toward social isolation. As mentioned in a related study, providing human care is currently becoming more difficult, and the development and introduction of socially assistive robots are often being considered to support people in social isolation in their daily lives by detecting the tendency toward cognitive decline.

However, the results of this study showed that it is difficult to recruit people who tend to be socially isolated by gathering research participants through methods such as posting. Since it is quite possible that the intervention effect of the socially assistive robots may vary depending on the study participants’ friendships, and if a function targeting users in social isolation is developed, the effectiveness of the method of recruiting participants should be carefully verified.

Because it is difficult to include older people in social isolation in an experiment, other information resources should be effectively utilized in the preliminary stages of recruiting participants for a study. For example, if there is a volunteer position for inclusive care in a community in Japan, they have information on households in social isolation and may be able to establish a cooperative relationship. In addition, ambient sensors in smart homes track the amount of activity of residents, and analysis of sensor information may indirectly help estimate isolation status.

### 6.3. Diligence

The diligence rate among workshop participants was 4.43, almost the same as the Japanese average for older people, 4.37. This rate may be due to the fact that the workshop lasted only two hours, which allowed study participants to register easily. On the other hand, the diligence of participants by staff selection was 4.72, which was 0.35 higher than the Japanese elderly average. This rate indicates that the facility staff recommended highly industrious people in terms of data acquisition for ongoing research.

Although negative differences in the population are generally perceived, there are also positive aspects. For example, some findings can only be obtained by taking long-term data, such as changes in the interactions between humans and robots over time. In the case of a long-term study involving a socially assistive robot intervention of several weeks to several months, a low dropout rate may be achieved with a diligent population. Therefore, implementing a Big-Five test is useful in finding a highly industrious population.

## 7. Conclusions

In this research, we compared the average personality characteristics of participants in a workshop on robotics recruited directly through posting with personality characteristics of older Japanese adults to examine participant selection bias and group representativeness for future study of SARs.

After a weeklong recruitment period, the workshop was attended by 20 older participants (nine males and 11 females) aged between 62 and 86 years. Extroversion among workshop participants was 4.38, 0.40 higher than the average for older adults in Japan. The workshop participants’ openness was 4.55, 1.09 higher than the average for the Japanese elderly. Thus, the results indicate a slight selection bias in the personal characteristics of the participants depending on the recruitment method when compared to the national average.

Also, only one of 20 participants was below the cutoff on the LSNS-6 score and was considered to have a tendency toward social isolation. The development and introduction of socially assistive robots are often considered to support people in social isolation in their daily lives, however, the results of this study showed that it is difficult to recruit people who tend to be socially isolated when gathering research participants by methods such as posting. Therefore, the effectiveness of the method of recruiting participants should be carefully verified in the research regarding socially assistive robots.

Overall, we demonstrated the risk of introducing bias in the personality traits of the participants depending on the participant recruitment method, as well as the population representativeness of the selected sample. Our suggested countermeasure is increasing the sample size as much as possible and conducting a stratified analysis for each personality trait. Alternatives include using other information resources in the preliminary stages of recruiting participants, such as referring to a member of the community welfare committee for comprehensive supportive care and drawing on sensor analysis information in smart homes in Japan.

## Figures and Tables

**Table 1:** Eight items for (2) the impressions of the life support robots.

(1) Do you feel at ease with this robot? (2) Does this robot make you feel relieved? (3) Do you think this robot will heal you? (4) Do you feel secure with this robot? (5) Does the way this robot moves feel familiar? (6) Does this robot look friendly? (7) Do you feel comfortable with this robot? (8) Does the way this robot reacts feel gentle?

**Table 2:** Comparison of Personality Scores of Workshop Participants, Staff Selection Participants, and National Average.

	Japanese Average (a)	Staff Selection Participants’ Average (b)	Workshop Participants’ Average (c)	Difference (b−a)	Difference (c−a)	Difference (b−c)
Extroversion	3.95	4.78	4.38	0.83	0.43	0.40
Agreeableness	5.63	5.78	5.48	0.15	−0.15	0.30
Conscientiousness	4.37	4.72	4.43	0.35	0.06	0.29
Neuroticism	4.13	3.56	3.13	−0.57	−1.00	0.43
Openness	3.46	4.33	4.55	0.87	1.09	−0.22

**Table 3:** Comparison of correlation coefficients between interest in experimenting with robots in the home and other factors.

	Mean	Standard Deviation	Correlation Coefficient	*n*
Interest in experimenting with robots in the home	3.30	0.92	n/a	20
Age	74.55	5.54	−0.10	20
Impression of communication robots	5.14	0.68	0.38	20
LSNS-6	17.65	3.77	−0.29	20
Extroversion	4.38	1.06	−0.23	20
Agreeableness	5.48	0.82	−0.20	20
Conscientiousness	4.43	1.32	−0.07	20
Neuroticism	3.13	1.19	0.18	20
Openness	4.55	1.33	−0.21	20

## Data Availability

The data that support the findings of this study are available from the corresponding author upon reasonable request.

## References

[1] Ministry of Health, Labor and Welfare (2018). Annual Report for Aging Society.

[2] Ministry of Health, Labor and Welfare (2018). Required Number of Care Personnel Based on the 7th Care Insurance Plan.

[3] Nihei M., Inoue T., Nishiura Y., Mamiya I., Onaka S., Watabe K., Osawa Y., Shimizu Y., Harada A., Hiroaki K. (2015). Relaxed watching system for elderly by using communication robot. JSMBE.

[4] Alonso S.G., Hamrioui S., Díez I.D.L.T., Cruz E.M., López-Coronado M., Franco-Martin M. (2019). Social Robots for People with Aging and Dementia: A Systematic Review of Literature. Telemed. e-Health.

[5] Chen S., Jones C., Moyle W. (2018). Social Robots for Depression in Older Adults: A Systematic Review. J. Nurs. Scholarsh..

[6] Igarashi T., Nihei M., Nakamura M., Obayashi K., Masuyama S., Kamata M. Socially assistive robots influence for elderly with cognitive impairment living in nursing facilities: Micro observation and analysis. Proceedings of the 15th AAATE Conference 2019.

[7] Vandemeulebroucke T., de Casterlé B.D., Gastmans C. (2016). How do older adults experience and perceive socially assistive robots in aged care: A systematic review of qualitative evidence. Aging Ment. Health.

[8] Feil-Seifer D., Mataric M.J. Defining Socially Assistive Robotics. Proceedings of the 9th International Conference on Rehabilitation Robotics 2005.

[9] Sakuma M. (2018). Current status and issues of interaction to communication robots: Focusing on negative feeling. Bull. Shiraume Gakuen Univ. Jr. Coll..

[10] Eiki T. (2017). Fundamentals of research design and statistical analysis. Physiotherapy.

[11] Martinson B.C., Crain A.L., Sherwood N.E., Hayes M.G., Pronk N.P., O’Connor P.J. (2010). Population Reach and Recruitment Bias in a Maintenance RCT in Physically Active Older Adults. J. Phys. Act. Health.

[12] Corr P.J., Gerald M. (2009). The Cambridge Handbook of Personality Psychology.

[13] Goldberg L.R. (1990). An alternative description of personality: The big-five factor structure. J. Personal. Soc. Psychol..

[14] Hofstee W.K., De Raad B., Goldberg L.R. (1992). Integration of the big five and circumplex approaches to trait structure. J. Personal. Soc. Psychol..

[15] McCrae R.R., Costa P.T. (1987). Validation of the five-factor model of personality across instruments and observers. J. Personal. Soc. Psychol..

[16] Dollinger S.J., Leong F.T. (1993). Volunteer Bias and the Five-Factor Model. J. Psychol. Interdiscip. Appl..

[17] Briggs S.R. (1992). 1992 Assessing the five−factor model of personality description. J. Pers..

[18] Baumeister R.F., Gailliot M., DeWall C.N., Oaten M. (2006). Self-regulation and personality: How interventions increase regulatory success, and how depletion moderates the effects of traits on behavior. J. Personal..

[19] Crocker J., Canevello A. (2008). Creating and undermining social support in communal relationships: The role of compassionate and self-image goals. J. Personal. Soc. Psychol..

[20] Shinji K., Satoshi A., Cutrone P. (2012). Attempt to create Japanese version of Ten Item Personality Inventory (TIPI-J). Personal. Res..

[21] Hajime I., Yuko Y. (2018). Examination of standard value and sex difference and age difference of Japanese version Ten-Item Personality Inventory (TIPI-J) in middle-aged and elderly people. Jpn. Public Health J..

[22] Igarashi T., Nihei M., Mizuno J., Inoue T., Kamata M. (2019). Subject selection bias in intervention experiments with socially assistive robots and the impact on the representativeness of the population. Social Robotics. Proceedings of the 11th International Conference, ICSR 2019.

[23] Chang Q., Sha F., Chan C.H., Yip P.S. (2018). Validation of an abbreviated version of the Lubben Social Network Scale (LSNS-6) and its associations with suicidality among older adults in China. PLoS ONE.

[24] Kurimoto A., Awata S., Ohkubo T., Tsubota-Utsugi M., Asayama K., Takahashi K., Suenaga K., Satoh H., Imai Y. (2011). Creation and Reliability and Validity of the Japanese Lubben Social Network Scale Short Version (LSNS-6). J. Jpn. Geriatr. Soc..

[25] Sayuri Y., Hiroki Y. (2015). Social Isolation of Elderly People in Need of Assistance at Home and Its Related Factors. Bull. Niimi Public Univ..

[26] Kamide H., Arai T. (2021). Caring for Things Helps Humans Grow: Effects of Courteous Interaction with Things on Pro-Environmental Behavior. Sustainability.

[27] https://www.necplatforms.co.jp/solution/papero_i/index.html.

